# Editorial: Non-invasive physiological measurements: from discovery to implementation

**DOI:** 10.3389/fphys.2023.1356664

**Published:** 2024-01-11

**Authors:** Gabriel Zieff, Lauren C. Bates-Fraser, Patricia Pagan Lassalle, Craig Paterson, Kevin Heffernan, Lee Stoner

**Affiliations:** ^1^ Department of Exercise and Sport Science, University of North Carolina at Chapel Hill, Chapel Hill, NC, United States; ^2^ School of Kinesiology, University of British Columbia, Vancouver, BC, Canada; ^3^ Department of Epidemiology, The Gillings School of Global Public Health, University of North Carolina at Chapel Hill, Chapel Hill, NC, United States; ^4^ David B. Falk College of Sport and Human Dynamics, Syracuse, NY, United States; ^5^ Department of Biobehavioral Sciences, Teachers College, Columbia University, New York, NY, United States

**Keywords:** non-invasive measurements, physiological measurements, discovery, clinical, translation

## Introduction

The goal of this Research Topic, developed by the American College of Sports Medicine Non-Invasive Physiology (ACSM-NIP) interest group, was to demystify and increase the accessibility of non-invasive physiological measurement tools and procedures. The nomenclature “noninvasive physiological measurement” typically conjures imagery of laboratory wizardry and blue-skies science (e.g., where real-world applications are not immediately apparent). However, compared to invasive measurements, noninvasive physiological measurements often reduce financial and time burdens, lower risk of harm, and provide greater accessibility. These benefits may extend beyond the research setting to the clinical setting. Non-invasive measurements are crucial to a myriad of study types and research questions with implications spanning the research-clinical spectrum. Broad examples of research types in which non-invasive physiological measurements are used or have important implications include: i) discovery (interpreting clinical signals to better understand systems physiology), ii) clinical and preclinical/subclinical (establishing efficacy and safety of techniques or interventions, iii) epidemiological: identifying the distribution of disease or track cohorts, and iv) implementation (medical system approaches for tracking patient health). We, via this Research Topic, were interested in all article types (e.g., original research, brief reports, etc.) including those focused on bioengineering, mathematical modelling, laboratory-studies, clinical studies, epidemiological studies, and implementation studies. We were also interested in articles with implications for diversity, equity, and inclusivity, including those addressing life-stage, sex, gender, race, ethnicity, and disabilities.

Below we summarize the articles that were accepted for publication as part of this Research Topic. The included manuscripts, which will be described below, broadly fit into two main categories: i) development of tools to assess vascular function, and ii) strategies to assess and enhance functioning among individuals experiencing physical consequences of neurological, cerebral, and/or cognitive decrements.

## Theme 1: development of vascular methods

### Article summaries

Four submissions to this Research Topic focused on the development of novel tools to assess different components of vascular structure and function, ranging from the macro-vascular level (e.g., assessments of arterial structure and function) to the micro-vascular level (e.g., assessments of capillary and mitochondrial function). At the macro-level, three contributions looked at techniques related to the measurement of arterial stiffness, a composite measure of arterial structure and function. First, ballistocardiography was used by Steffensen et al. as a novel tool to measure pulse transit time (PTT). More specifically, they used a wrist-worn ballistocardiogram (BCG) to assess PTT during acute exercise cycling at differing intensities and postures (Steffensen et al.). Their findings showed that there were differences in PTT across different postures and intensities. Echocardiography also confirmed the association of BCG features with aortic valve opening as well as a positive relationship between BCG amplitude and stroke volume (Steffensen et al.).

Zieff et al. examined a novel strategy to assess PWV, another measurement related to arterial stiffness. Using a modified head-up tilt test (mHUTT) as an acute physiological perturbation, they tested the agreement between a simple photoplethysmography (PPG)-based PWV strategy with a criterion oscillometric method, including measurement in both the upper- and lower-limbs (Zieff et al.). Agreement between the devices, including in terms of the change in PWV elicited by the mHUTT, was only acceptable in the lower, but not the upper-limbs. Nevertheless, findings showed that PPG may be a feasible strategy to continuously assess PWV (Zieff et al.).

D’Agata et al. published a perspective piece on the passive leg movement technique, an assessment of lower-limb arterial function that is conducted using Doppler ultrasound to determine leg blood flow. The PLM technique is thought to reflect nitric-oxide mediated endothelial function, a crucial physiological function related to atherosclerosis and early stages of vascular disease. In their perspective piece, they discuss and demonstrate the feasibility of performing PLM in children and adolescents, and provide preliminary PLM-induced leg blood flow values from their laboratory in children and adolescents.

At the micro-vascular level, Hiura et al. investigated the use of near-infrared spectroscopy (NIRS) to investigate the relationships between cerebral oxygen metabolism and perfusion in the prefrontal cortex during different exercise intensities. They demonstrated dissociated coupling between cerebral oxidative metabolism and perfusion in the pre-frontal cortex (PFC) during the transition from light to moderate exercise as well as after the cessation of exercise (Hiura et al.). This work represents the first preliminary findings regarding the decoupling between cerebral oxidative metabolism and perfusion in the prefrontal cortex using NIRS during exercise.

### Lessons learned

Despite being modifiable through lifestyle factors (e.g., physical activity, diet, sleep, etc.) cardiovascular diseases (CVD) continue to be the leading cause of death globally. CVD risk begins early in the life course (e.g., childhood, adolescence, young-adulthood), often decades or years before overt or clinical symptoms arise. The types of non-invasive vascular assessments represented in the Research Topic reflect a promising research area with the potential to improve prognostic and clinical comprehension and detection of early, potentially modifiable stages of vascular pathophysiology.

One way in which these techniques may facilitate the investigation of the importance of risk factors to vascular physiology is by having the capacity to assess changes to acute physiological or environmental perturbations. For example, static markers confer limited pathophysiological insight, whereas reactivity to acute “stressors” reflect the functional capacity of vascular systems and sub-systems. Acute exercise is one common way to elicit a physiological challenge to the cardiovascular (CV) system. As part of this Research Topic, Hiura et al. and Steffensen et al. both examined the impact of acute exercise on different physiological changes. The findings by Hiura et al. demonstrate a non-invasive measurement technique that reflects acute changes in cellular-level cardiometabolic alterations in response to exercise. Their technique’s ability to capture responses to exercise enabled the detection of the real-time regulation of the relationship between cerebral oxygen metabolism and perfusion in the PFC. These findings highlight the functional role of the PFC during exercise which may have implications for better understanding the inter-relationships between lifestyle factors and cerebrovascular diseases including those affecting cognitive function.

The wrist BCG investigated by Steffensen et al. was also tested in response to an acute bout of exercise. Their findings demonstrated a convenient assessment of the transit time of pressure waves through the arterial tree (i.e., PTT) during acute exercise. Their findings may also better enable studies of CV response to acute exercise and improve the feasibility of continuous, cuffless monitoring of blood pressure and CV performance. Since PTT is a constituent component of PWV estimations, the ability to assess PTT also enhances the possibility for the development of novel measurements of PWV such as the method described by Zieff et al.

Zieff et al. used the mHUTT rather than an acute exercise bout to challenge the vascular system (Zieff et al.). Their technique tested a novel PPG system’s ability to capture the physiological reactivity to a postural shift-induced autonomic challenge (Zieff et al.). Their findings showed that PPG is a feasible strategy to continuously assess limb-specific PWV, though agreement was only acceptable in the lower- and not the upper-limb (Zieff et al.). One of the major take-aways from their findings, similar to the BCG technique described by Steffensen et al., was the feasibility for continuous, cuffless measurement of PWV, which better enables accurate reflections of acute vascular reactivity and function. Improved ability to monitor these acute changes will aid in elucidating how acute exposures to modifiable lifestyle factors important to CV health (e.g., prolonged sitting, consuming a high-sugar snack, etc.) may contribute to CVD risk over time.

The findings from this Research Topic also highlight important implications for the assessment of vascular function in understudied, yet important populations. D’Agata et al.’s review demonstrates the feasibility of performing PLM to assess vascular and endothelial function in children and adolescents. The pediatric obesity epidemic and rising rates of type II diabetes development among children and adolescents supports the need for feasible tools such as the ultrasound-based PLM technique to assess vascular health in these early life stages when pathophysiological changes begin to occur within the vasculature. If we can identify physiologically “normal” *versus* “abnormal” changes in pediatric and adolescent vascular systems, we may be better able to intervene sooner, and thereby reduce the burden of disease later in life.

## Theme 2: neuro/cerebro/cognition and physical functioning

### Article summaries

Two studies explored the use of novel approaches for monitoring and enhancing functioning among individuals experiencing physical consequences of neuro-cognitive decline. Faulkner et al. sought to identify whether a home-based overground robotic gait training technology–when used in conjunction with usual care physical therapy–enhances vascular health among chronic stroke patients. They found that PWV was reduced (improved) to a greater exent in the patients randomized to receive the robotic gait-training (Faulkner et al.). These improvements were likely mediated by increases in physical activity and decreases in sedentary behavior, which were assessed using accelerometry during a 7-day free living period. Especially noteworthy, the PWV improvements observed in the robotic gait-training group were maintained 3 months after completing the gait training program (Faulkner et al.).

Pereira and Hunter contributed a review centered on the utility of complementing physical tasks with cognitive tasks to more accurately assess motor function. Their review highlighted that tasks requiring simultaneous physical activity and cognitive challenges resulted in greater reductions of important functional characteristics of motor performance compared to isolated physical activity tasks. Because of this greater challenge imparted by the simultaneous physical activity and cognitive tasks, the authors argue that these simultaneous tasks are better able to illuminate disturbances and differences in motor performances across varying populations. For example, simultaneous cognitive and physical tasks exacerbated the reductions in motor performance in older adults, especially females (Pereira and Hunter). The authors further comment on the central and peripheral mechanisms at play which likely underlie age- and sex-related decrements in motor performance (Pereira and Hunter).

### Lessons learned

Diminished capacity to function and perform physically occurs naturally with aging. Neurological and cerebrovascular decline (and disease states), in addition to their intrinsic negative effects on cognitive function, represent common sources of worsening physical functioning during aging. With the number of people aged 60+ expected to double worldwide from 2015 (12%) to 2050 (22%) (WHO, 2023), there is a critical need to determine best practices for monitoring, understanding, and minimizing the loss of physical functioning which accompanies aging and neurological decline. The contributions from Faulkner et al. and Pereira et al. both address this critical need.

The review by Pereira and Hunter supports the utility of a novel paradigm to challenge and assess motor function. The evidence presented in their review has important implications for understanding motor function in at-risk and clinical populations. More specifically, the utilization of a simultaneous cognitive and physical task may more accurately challenge the neuromuscular system in future research designed to better understand pathophysiological progression and elucidate intervention targets for age- and disease-related physical decline. Possible populations for which this paradigm may be relevant include but are not limited to geriatric populations and Alzheimer’s, Parkinson’s, and stroke patients–the latter being the same population investigated in the study by Faulkner et al.

Stroke is a cerebrovascular disease which results in neurological, cognitive, and motor disturbances and is often accompanied by a rapid loss in the ability to perform activities of daily living. Faulkner et al. demonstrated that the robotic gait training in addition to physical therapy was an effective strategy to improve activity-behaviors and reduce arterial stiffening among a clinical stroke population. It is certainly important to acknowledge that robotic gait-training likely requires access to special resources and may be cost-prohibitive for many individuals. Nevertheless, findings from Faulkner et al. do highlight that technology-assisted movement may be a promising strategy to ameliorate the physical deconditioning and elevated CVD risk associated with stroke. Their implementation of this technique within an unsupervised home-setting further underscores its potential feasibility at scale.

## Conclusion

Non-invasive physiological measurements play a vital role in assessment of many physiological systems and across a range of research applications from discovery to clinical implementation. The goal of this Research Topic, developed by the ACSM-NIP interest group, was to demystify and increase the accessibility of non-invasive physiological measurement tools and procedures. The six articles included in this Research Topic have important implications for monitoring sub-clinical progression of CVD risk and using novel tools to better understand and help mitigate the negative effects of neuro-cognitive and cerebrovascular disease on physical function and health. Non-invasive physiological measurement tools and procedures have the potential to enhance research and clinical practices while minimizing participant (and patient) burden. Expanding and implementing knowledge surrounding non-invasive measurement methodologies can play an important role in combating global health challenges including CVD and neurological disorders.
